# Reconstruction of a Complex Anterior Maxillary Defect Using the Bone‐to‐Soft Tissue Technique: A Case Report

**DOI:** 10.1155/crid/6114010

**Published:** 2026-06-24

**Authors:** Boris Baruch S. Bernatskiy, Joseph Y. Kan, Ekaterina A. Zernitckaia

**Affiliations:** ^1^ Independent Researcher, Moscow, Russia; ^2^ Advanced Educational Program in Implant Dentistry, Loma Linda University School of Dentistry, Loma Linda, California, USA, llu.edu

**Keywords:** alveolar ridge reconstruction, bone-to-soft tissue technique, dental trauma rehabilitation, dentoalveolar transplantation, esthetic zone reconstruction, tooth autotransplantation

## Abstract

**Background:**

Reconstruction of alveolar defects in the esthetic zone remains challenging, particularly when both hard‐ and soft‐tissue deficiencies coexist or when implant placement is contraindicated due to age‐related or anatomical factors. Biologically driven approaches aiming to simultaneously restore the dentoalveolar unit have gained increasing interest.

**Case Presentation:**

This case report describes the clinical application of a Bone‐to‐Soft Tissue (B2S) protocol involving transplantation of a combined tooth–bone–soft‐tissue complex in a 17‐year‐old patient presenting with traumatic loss of a maxillary central incisor and severe alveolar ridge deficiency. The surgical procedure included harvesting of the dentoalveolar complex, adaptation to the recipient site, stabilization, and staged restorative management.

**Results:**

Stable integration of the transplanted complex was achieved, with preservation of soft‐tissue architecture and restoration of alveolar contour. Radiographic follow‐up confirmed maintenance of bone volume, whereas clinical outcomes demonstrated satisfactory esthetic and functional rehabilitation.

**Conclusions:**

Composite tooth–bone–soft‐tissue transplantation within the B2S technique may represent a biologically oriented alternative for selected cases of esthetic zone reconstruction, particularly when conventional implant or regenerative approaches are limited. Further controlled studies are required to validate long‐term outcomes and define clinical indications.

## 1. Introduction

Reconstruction of alveolar defects in the esthetically critical zone remains one of the most demanding challenges in contemporary implantology and periodontology. Achieving stable hard‐ and soft‐tissue architecture while maintaining natural esthetics requires highly predictable biological approaches. Despite significant advances in regenerative techniques and implant therapy, complications such as soft‐tissue instability, gingival recession, and progressive bone remodeling continue to affect long‐term outcomes.

These challenges are further amplified in younger patients, where ongoing craniofacial growth must be considered. Dental implants behave as ankylosed structures and do not adapt to physiological dentoalveolar development, which may result in infraocclusion and progressive esthetic disharmony over time [1]. Moreover, dentoalveolar adaptation has been shown to persist even after skeletal maturity, with continuous eruption of maxillary incisors contributing to changes in crown length and gingival position [2, 3]. As a result, implant‐based rehabilitation may remain suboptimal not only in adolescents but also in certain adult patients.

At the same time, traumatic dental injuries frequently affect young individuals and represent a major cause of anterior tooth loss [4–7]. This creates a clinical dilemma in which definitive implant therapy may need to be postponed, whereas functional and esthetic rehabilitation is still required.

Conventional treatment options, including implant‐supported restorations, orthodontic space closure, adhesive prostheses, and guided bone regeneration (GBR), each present inherent limitation. Implant therapy often requires staged augmentation procedures and does not accommodate physiological tissue adaptation. Autotransplantation offers a biologically favorable solution for tooth replacement [8–11]. Variations of dentoalveolar and en bloc transplantation approaches have also been described for management of impacted teeth and localized alveolar reconstruction [12]. However, these techniques remain limited in their ability to predictably restore the complete dentoalveolar unit in severe anterior defects. GBR or autogenous bone block augmentation may restore hard tissue volume but typically involve multiple surgical stages, prolonged healing, and unpredictable vertical bone gain, particularly in relation to adjacent teeth [13, 14]. Furthermore, these approaches do not simultaneously restore the soft‐tissue component of the dentoalveolar unit. Taken together, these limitations highlight the ongoing challenge of achieving simultaneous restoration of the entire dentoalveolar unit, including bone, soft tissue, and the tooth, within a single surgical intervention.

The Bone‐to‐Soft Tissue (B2S) concept represents a biologically oriented approach that includes several reconstructive protocols aimed at combined hard‐ and soft‐tissue restoration while preserving anatomical relationships [15]. Among these, transplantation of a combined tooth–bone–soft‐tissue complex constitutes one specific clinical application. The present report expands upon the previously introduced B2S concept by illustrating its application in reconstruction of a severe anterior maxillary defect associated with traumatic tooth loss and alveolar ridge deficiency in a growing patient.

A key biological aspect of this approach is the preservation of periodontal ligament vitality, which may support revascularization, functional adaptation, and integration of the transplanted complex within the recipient site. In contrast to conventional graft‐based procedures, this may contribute to more stable tissue architecture within a physiologic environment. Within these boundaries, the B2S approach may be considered a potential treatment option in selected clinical situations, including traumatic tooth loss, postimplant complications, or cases in which implant placement is limited by anatomical or age‐related factors.

The present case report describes the clinical application of combined tooth–bone–soft‐tissue complex transplantation in a 17‐year‐old patient with traumatic loss of a maxillary central incisor and severe alveolar ridge deficiency, illustrating surgical principles and short‐ to mid‐term clinical and radiographic outcomes.

## 2. Case Presentation

This report describes a single clinical case and did not involve a systematic investigation designed to produce generalizable knowledge; therefore, formal Institutional Review Board approval was not required according to institutional policy. The case was conducted in accordance with the principles of the Declaration of Helsinki. Written informed consent for treatment, publication of clinical data, and use of clinical photographs and radiographic images was obtained from the patient. All identifying personal information was omitted to protect patient anonymity.

### 2.1. Extra‐ and Intraoral Examination

A 17‐year‐old patient was referred to the clinic presenting with an esthetic anterior defect. Dental history revealed traumatic loss of the right maxillary central incisor at the age of 11 (Figure 1A, B). At the age of 14, the missing tooth was replaced with an adhesive (Maryland) bridge. Absence of socket preservation resulted in the development of a pronounced combined hard‐ and soft‐tissue defect.

**Figure 1:**
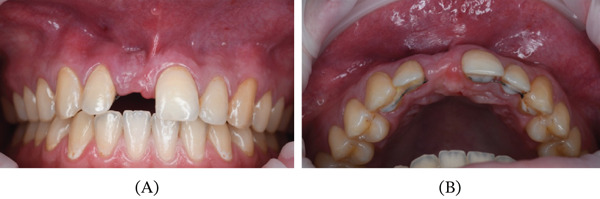
Initial clinical situation. (A) Buccal intraoral view. (B) Occlusal view.

Cone‐beam computed tomography (CBCT) (voxel size 0.2 mm) demonstrated severe buccal bone resorption in the projection of the right maxillary central incisor, with a coronal ridge width of 2.6 mm and an apical width of 5.6 mm (Figure 2A, B).

**Figure 2:**
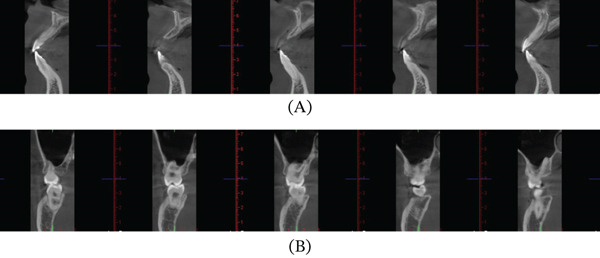
Preoperative CBCT assessment. (A) Recipient site demonstrating severe buccal bone resorption in the region of the right maxillary central incisor. (B) Donor site evaluation demonstrating favorable anatomical conditions for harvesting of the dentoalveolar complex.

Preoperative CBCT evaluation of the donor site demonstrated favorable anatomy for harvesting of the dentoalveolar complex. The palatal root of the right maxillary third molar measured approximately 10.6 mm in length, with a coronal width of 6.0 mm and an apical width of 3.2 mm. The soft‐tissue thickness in the maxillary tuberosity region distal to the third molar was approximately 6.3 mm, providing sufficient tissue volume for harvesting of the associated submucosal layer.

Clinical evaluation confirmed gingival recession on the right lateral incisor (Cairo Type 2 classification) combined with mild buccal and interproximal clinical attachment loss, a condition generally associated with a less favorable esthetic prognosis for implant therapy [16]. Periodontal examination of the adjacent teeth revealed shallow probing depths ranging from 1 to 3 mm, without bleeding on probing or tooth mobility. The patient presented with an average smile line and a clinically thick gingival phenotype based on probe transparency assessment, indicating a relatively low esthetic risk profile. Oral hygiene status was good (OHI‐S 0.7).

Considering the patient′s young age and ongoing craniofacial growth, alveolar ridge reconstruction was planned using transplantation of the right maxillary third molar as a dentoalveolar unit, harvested together with a submucosal soft‐tissue layer. A direct composite restoration was planned as a temporary solution with a full‐ceramic final crown.

### 2.2. Surgical Procedure

Considering the complexity of the reconstructive procedure and simultaneous transplantation of a dentoalveolar complex, systemic antibiotic prophylaxis was administered. Amoxicillin (875 mg) with clavulanic acid (125 mg) was prescribed 1 h prior to surgery and continued twice daily for 7 days.

Local infiltration anesthesia was induced using 5.1 mL (three carpules) of Ubistesin Forte (articaine hydrochloride 4% with epinephrine 1:100,000; 3 M Deutschland GmbH, Neuss, Germany) at the donor site (maxillary tuberosity and right maxillary third molar) and 3.4 mL (two carpules) at the recipient site.

### 2.3. Preparation of the Recipient Bed

Incisions were performed using a 15C blade (10‐256‐15, Hu‐Friedy, Mfg. Co., LLC, Frankfurt am Main, Germany). A crestal incision was shifted 3 mm palatally to facilitate suturing over the area occupied by the transplanted tooth. Intrasulcular incisions around adjacent teeth were made only on the buccal aspect and extended up to the distal line angles of the teeth bordering the defect. Vertical releasing incisions were initiated from the gingival margin and extended to the mucogingival junction in a hockey‐stick design, using a new 15C blade positioned at 90° to the bone (Figure 3).

**Figure 3:**
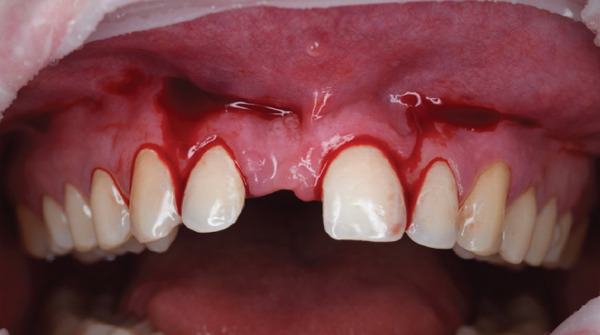
Incision design at the recipient site.

After elevation of a full‐thickness mucoperiosteal flap, the recipient bed was refreshed using a sickle scaler (EverEdge 2.0, SYG7/89E2, Hu‐Friedy Manufacturing Co., LLC, Chicago, IL, United States) and covered with a sterile gauze soaked in saline during graft harvesting from the donor site (Figure 4).

**Figure 4:**
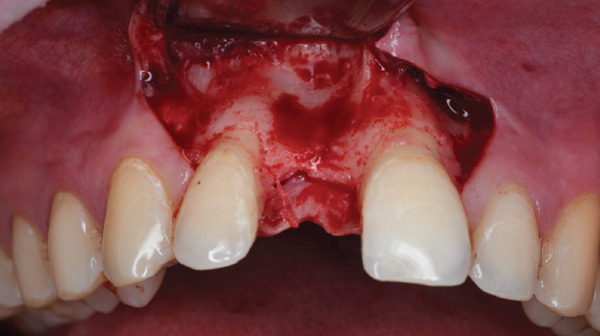
Full‐thickness mucoperiosteal flap elevation.

### 2.4. Donor Site and Graft Harvesting

An intrasulcular incision was made around the right maxillary third molar (15C blade 10‐256‐15, Hu‐Friedy, Frankfurt am Main, Germany). Periodontal ligament detachment was performed buccally with a periotome (PT1, Hu‐Friedy, Frankfurt am Main, Germany).

A split‐thickness palatal flap was then prepared through the sulcus of the third molar using a 15C blade inclined parallel to the palatal surface and continued apically toward the root apex. The incision between the second and third molar was positioned precisely in the middle of the interdental space, as it was essential to preserve sufficient bone over the distal surface of the second molar roots; this incision served as a reference line for the subsequent osteotomy.

Distal to the third molar, a straight crestal incision (#12 or #15C blade, Hu‐Friedy, Frankfurt am Main, Germany) was made in the midline of the tuberosity, dividing it into two equal parts.

Following completion of flap splitting, one mesial and one apical osteotomy were prepared using a piezoelectric surgical device (VarioSurg, NSK Nakanishi, Inc., Kanuma, Tochigi, Japan) operated at 100% power and irrigation settings, using straight (SG1) and angled (SG14L) surgical tips. The osteotomy between the second and third molar was initiated along the premade incision: starting at the midline of the third molar and extending palatally with a vertical cut down to the apex and 1–2 mm beyond. From this point, a horizontal cut was initiated perpendicular to the alveolar ridge and continued distally parallel to the tuberosity incision.

An angled 15‐mm flap chisel (1676‐08, Lexer Mini, A. Schweickhardt GmbH & Co. KG, Germany) was introduced into the osteotomies to create a controlled fracture, facilitating harvest of the third molar together with the bone block.

Forceps (FX67A, Hu‐Friedy, Mfg. Co., LLC, Frankfurt am Main, Germany) were applied to the crown with gentle rotation and luxation allowed extraction of the tooth together with the corticocancellous bone layer covered by a soft‐tissue component. Precise cuts and strict control of the harvesting procedure are essential to obtain the dentoalveolar unit in one piece. Very thin and meticulous dissection of the periodontal ligament helped preserve the sockets of the buccal roots intact (Figure 5).

**Figure 5:**
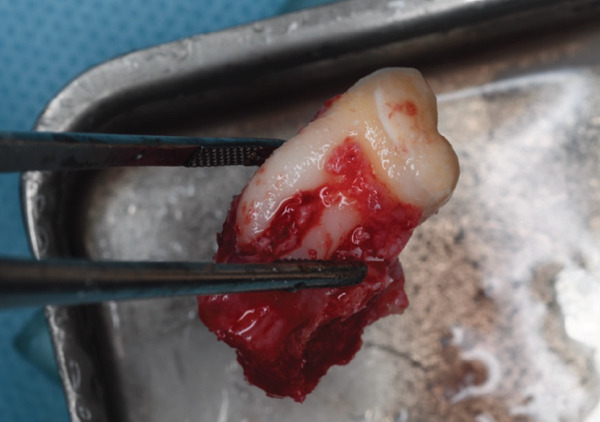
Harvested dentoalveolar complex.

### 2.5. Root Modification and Graft Adaptation

Hemisection of the buccal roots was performed extra‐orally with copious irrigation using a Lindemann bur (H162SXL.314.014, Komet Dental, Gebr. Brasseler GmbH & Co. KG, Lemgo, Germany) (Figure 6). Intraoral hemisection was not considered feasible due to limited access and visibility. Grinding rather than cutting movements of the bur were used to minimize the risk of damaging the surrounding bone and periodontal ligament structures. Throughout the extraoral manipulation, the transplanted complex was continuously wrapped in sterile saline‐soaked gauze to minimize dehydration and preserve periodontal ligament vitality.

**Figure 6:**
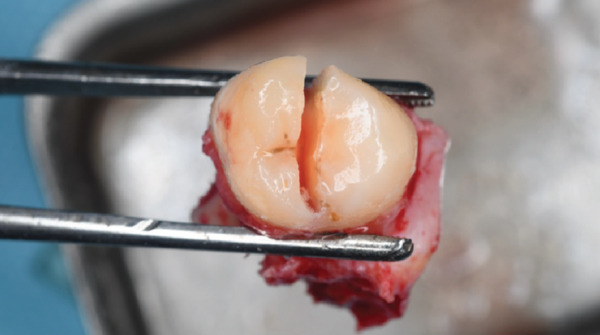
Extraoral root modification via hemisection.

The recipient site had been fully prepared prior to graft harvesting, allowing immediate transfer and adaptation of the transplanted complex without prolonged extraoral storage. Harvesting of the dentoalveolar complex required approximately 10 min, whereas extraoral root modification and graft preparation were completed within approximately 5 min before placement into the recipient bed.

Following hemisection, pulpal hemostasis was achieved using a collagen sponge. The exposed pulp chamber and perforation areas were then sealed with a calcium‐silicate–based bioactive cement (Biodentine, Septodont, Saint‐Maur‐des‐Fossés, France), selected to enhance adaptation within the modified root anatomy and support biologic sealing, and subsequently covered with a flowable composite resin (ENAMEL plus HRi, Micerium S.p.A., Avegno, Italy).

The furcation area of the transplanted palatal root was adjusted to the recipient bed to achieve intimate adaptation. The palatal root was selected as the primary transplanted root component due to its more favorable morphology and ability to provide greater structural support within the recipient site after hemisection. The attached corticocancellous bone segment was intentionally oriented buccally to compensate for the severe buccal ridge deficiency and restore the external contour of the alveolar process. Hemisection of the buccal roots was performed to reduce graft volume, improve adaptation to the narrow recipient site, and facilitate passive positioning of the dentoalveolar complex. The gingival zenith was adjusted to match the contralateral central incisor. Final passive adaptation between the transplanted complex and recipient bed was achieved by selective grinding of the recipient site as needed.

The graft was stabilized through the corticocancellous layer with a single 12‐mm B2S fixation pin (PIN‐B2S‐01.06, Art Stomus, Saint Petersburg, Russia; approved for clinical use in the Russian Federation, Registration Certificate No. РЗН 2022/18701) (Figure 7A, B). In situations where the crown height can increase susceptibility to nonaxial occlusal forces, additional fixation may be achieved using flowable composite.

**Figure 7:**
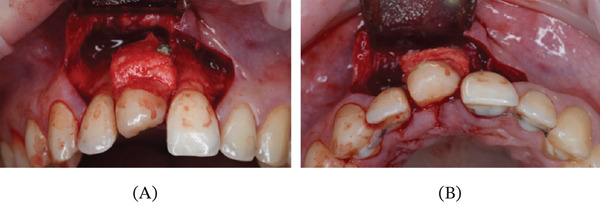
(A) Buccal view demonstrating fixation of the transplanted dentoalveolar complex using a B2S fixation pin. (B) Occlusal view.

Proper suturing is considered an additional stabilization factor for the transplanted unit. The palatal flap was sutured without flap advancement. For tension‐free closure and coronal advancement of the buccal flap, deep and superficial periosteal releasing incisions were performed (Figure 8).

**Figure 8:**
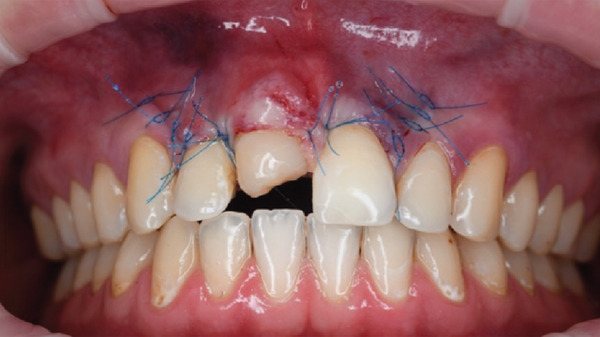
Flap closure and microsurgical suturing.

Microsurgical “Shanelec‐type” knot sutures were placed along the vertical releasing incisions [17]. Final tight flap adaptation and coronal seal were achieved with single interrupted sutures. A monofilament material (Prolene 6‐0; Ethicon, Johnson&Johnson MedTech, Raritan, NJ, United States) was used, as it is commonly preferred for esthetic‐zone procedures.

The donor site was sutured over a collagen sponge (Helistat, Integra LifeSciences Corporation, Princeton, NJ, United States) to support clot stabilization and soft‐tissue adaptation. Primary closure was achieved using tension‐free suturing. Sutures were removed on Postoperative Day 7. Donor site healing was uneventful and clinically comparable to conventional atraumatic maxillary third molar extraction healing. Follow‐up radiographic evaluation demonstrated progressive bone fill of the donor area, which became radiographically evident within approximately 4 months after surgery.

### 2.6. Endodontic Treatment

According to classical tooth autotransplantation protocols described by Tsukiboshi et al., root canal treatment is generally recommended approximately 2 weeks after transplantation, irrespective of root maturity, to reduce the risk of inflammatory resorption and pulpal complications [8]. However, when transplantation is performed as a dentoalveolar complex, postponement of endodontic intervention to approximately 4 weeks is advisable, since early endodontic manipulation may interfere with integration of the transplanted bone component. In the present case, consistent with the B2S protocol, endodontic treatment was therefore initiated on Postoperative Day 30, a time point typically associated with stable incorporation of the transplanted bone block and sufficient stabilization of the dentoalveolar unit (Figure 9).

**Figure 9:**
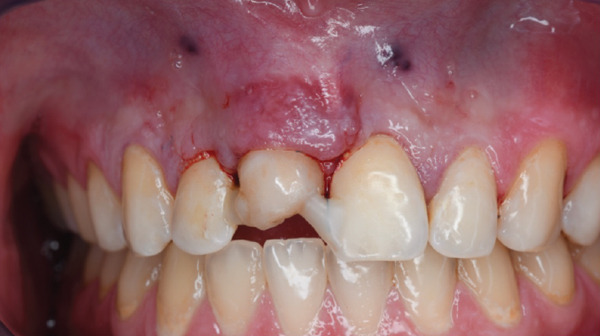
Early postoperative healing.

During the endodontic procedure, the crown of the transplanted dentoalveolar complex was additionally stabilized palatally using an orthodontic wire and interproximally with a flowable composite resin (ENAMEL plus HRi, Micerium S.p.A., Avegno, Italy) bonded to adjacent teeth following selective enamel spot etching and application of adhesive (OptiBond FL/Solo Plus, Kerr Dental, Orange, CA, United States). This stabilization minimized micromobility during instrumentation (Figure 10).

**Figure 10:**
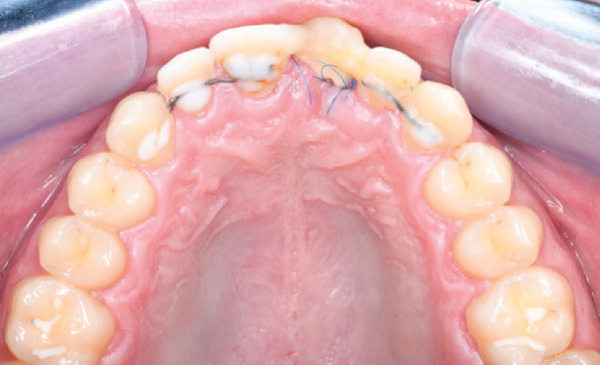
Palatal stabilization using an orthodontic wire.

After topical anesthesia with 10% lidocaine and infiltration anesthesia using articaine hydrochloride 4% with epinephrine 1:100,000 (Ubistesin Forte, 3 M Deutschland GmbH, Neuss, Germany), supragingival plaque removal was performed with AirFlow Plus powder (EMS Electro Medical Systems S.A., Nyon, Switzerland) in the anterior maxilla. The transplanted tooth was isolated with a rubber dam, and an access cavity was prepared.

The working length was determined radiographically. Root canal instrumentation was completed to size 35/0.04 using a nickel–titanium rotary system (Dentsply Maillefer, Ballaigues, Switzerland). Irrigation was performed using 3% sodium hypochlorite (3 mL per cycle) activated with passive ultrasonic irrigation (Irrisafe system, Satelec Acteon, Merignac‐Cedex, France). Ultrasonic activation was performed for 20 s over five irrigation cycles. Calcium hydroxide paste (UltraCal XS, Ultradent Products, Inc., South Jordan, UT, United States) was placed as an intracanal medicament for 2 weeks to reduce the risk of inflammatory root resorption. The access cavity was temporarily sealed with zinc oxide–eugenol cement (IRM, Dentsply Sirona, Charlotte, NC, United States) (Figure 11).

**Figure 11:**
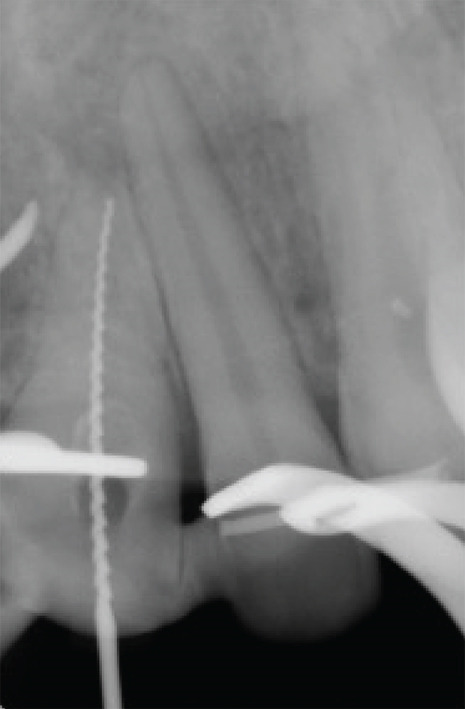
Initial endodontic stage.

Two weeks later, definitive endodontic treatment was completed. After repetition of anesthesia, professional plaque removal, rubber dam isolation, and removal of the temporary restoration, the canal was irrigated again with ultrasonically activated 3% sodium hypochlorite followed by 17% EDTA. Definitive obturation of the pulp chamber was performed using a bioactive dentin substitute (Biodentine, Septodont, Saint‐Maur‐des‐Fossés, France) (Figure 12).

**Figure 12:**
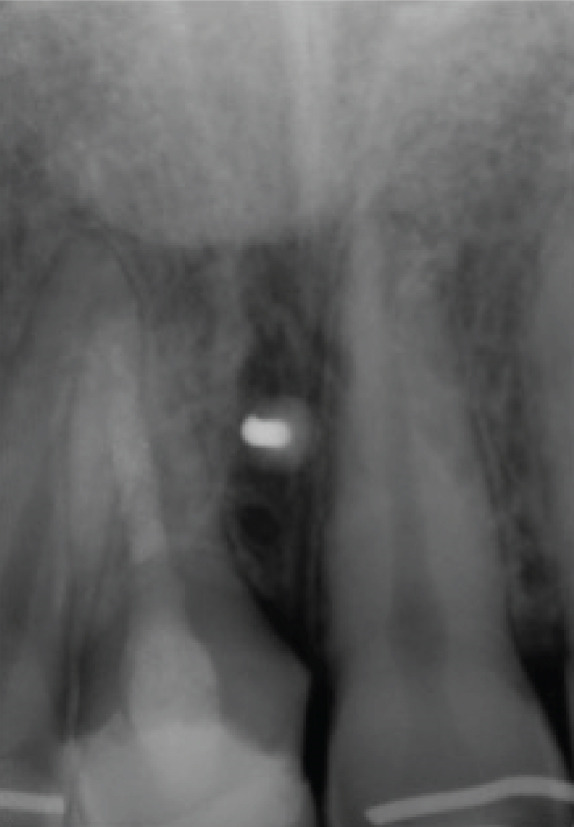
Completion of endodontic treatment. Final obturation.

The access cavity was subsequently treated with 27‐*μ*m aluminum oxide air abrasion, followed by adhesive application (OptiBond Solo Plus, Kerr Dental, Orange, CA, United States) and permanent composite restoration using ENAMEL plus HRi (Micerium S.p.A., Avegno, Italy).

At the 6‐month follow‐up, clinical evaluation demonstrated stability of the transplanted tooth–bone–soft tissue complex, with preservation of soft‐tissue architecture and absence of mobility or bleeding on probing (Figure 13A, B). Probing depths at the adjacent teeth remained within physiologic limits. CBCT evaluation confirmed maintenance of the augmented ridge dimensions, with ridge widths measuring 7.6 mm coronally, 10.2 mm at the midroot level, and 9.6 mm apically.

**Figure 13:**
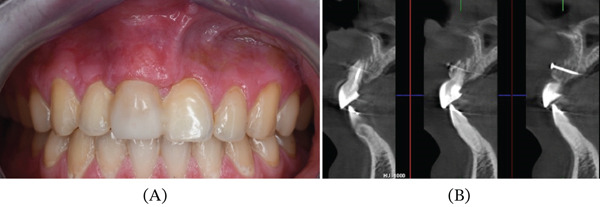
Six‐month follow‐up evaluation. (A) Clinical intraoral view demonstrating stable soft‐tissue architecture and satisfactory esthetic integration. (B) CBCT imaging confirming maintenance of bone volume and graft integration.

At the 3‐year follow‐up, the clinical findings remained stable, with preservation of gingival contours, absence of mobility, and healthy periodontal conditions around the adjacent teeth (Figure 14A–C). CBCT imaging demonstrated maintenance of the reconstructed alveolar ridge contour extending beyond the initial bony envelope. Ridge width measurements at the 3‐year follow‐up were 7.3 mm coronally, 12.3 mm at the midroot level, and 8.6 mm apically.

**Figure 14:**
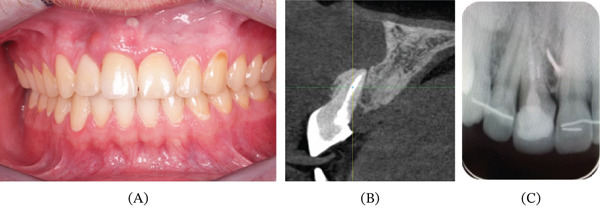
Three‐year follow‐up. (A) Intraoral clinical view demonstrating stable gingival contours and functional restoration. (B) CBCT imaging demonstrating maintenance of reconstructed bone volume. (C) Periapical radiograph confirming stable periodontal and periapical conditions.

## 3. Discussion

Reconstruction of complex defects in the esthetic zone requires treatment strategies capable of addressing both hard‐ and soft‐tissue deficiencies while ensuring long‐term functional and esthetic stability. The present case illustrates the application of dentoalveolar transplantation within the B2S concept as a biologically oriented approach for simultaneous reconstruction of the dentoalveolar unit in a challenging clinical scenario.

Conventional treatment modalities for anterior tooth loss each present important limitations when applied to cases with severe tissue deficiency. Implant‐supported restorations, although widely considered the standard of care, may be contraindicated or suboptimal in younger patients due to ongoing craniofacial growth and the ankylotic behavior of implants, which prevents physiological adaptation and may lead to infraocclusion over time [18, 19]. In addition, implant therapy in such cases often requires staged augmentation procedures, including GBR or bone block grafting, associated with prolonged treatment timelines and increased surgical burden.

From a reconstructive standpoint, vertical augmentation remains particularly challenging. The achievable bone height is often limited by adjacent bony peaks, whereas the use of barrier membranes requires a safety distance from neighboring teeth, frequently resulting in incomplete defect fill in these areas. Moreover, augmentation procedures typically necessitate subsequent soft‐tissue management to restore vestibular depth and mucogingival relationships.

Alternative nonsurgical or minimally invasive approaches, including adhesive bridges and orthodontic space closure, may provide acceptable functional outcomes but do not address the underlying deficiency of alveolar bone and soft tissue [20–23]. In addition, these approaches may compromise adjacent teeth or be limited in cases with extensive defects.

Tooth autotransplantation represents a biologically favorable treatment modality with high reported success rates [24–27], offering advantages such as preservation of periodontal ligament vitality, proprioception, and potential for orthodontic movement. However, conventional autotransplantation primarily addresses replacement of the missing tooth and typically requires an intact or minimally compromised recipient site. In cases of severe alveolar deficiency, the transplanted tooth alone may not provide sufficient reconstruction of the surrounding bone and soft tissue.

En bloc dentoalveolar transplantation has also been described, with reported effectiveness approaching 89% [12, 28]. However, this approach differs in its primary indication, as it is generally used for repositioning impacted teeth and may offer limited control over the bone component due to restricted surgical visibility.

Within this context, the B2S dentoalveolar transplantation concept introduces a reconstructive strategy by enabling transfer of a composite tooth–bone–soft‐tissue complex specifically designed to restore the dentoalveolar unit in a deficient site. This approach combines several key advantages. First, it allows simultaneous reconstruction of both hard and soft tissues in a single surgical procedure, reducing the need for staged interventions. Second, preservation of the periodontal ligament plays a critical role in maintaining vascular supply, facilitating revascularization, and reducing the risk of ankylosis. Third, transplantation of a vascularized bone component enables more rapid and physiologic remodeling compared with nonvascularized graft materials, potentially minimizing volumetric loss. Finally, the presence of an integrated soft‐tissue component contributes to improved stability of the gingival architecture and esthetic outcomes.

The radiographic findings observed in the present case further support the biological rationale of the B2S approach. A substantial increase in ridge dimensions was achieved and maintained over time, with coronal ridge width increasing from 2.6 mm preoperatively to 7.3 mm at the 3‐year follow‐up, whereas the apical width increased from 5.6 to 8.6 mm. Unlike conventional grafting procedures, where partial resorption and volumetric reduction are commonly observed, the relative stability of the reconstructed ridge in this case suggests favorable long‐term integration of the transplanted dentoalveolar complex. Although the exact mechanism underlying this finding cannot be determined from a single clinical case, preservation of periodontal ligament vitality and physiologic remodeling associated with the transplanted complex may have contributed to the observed dimensional stability. In addition, ongoing dentoalveolar adaptation related to residual craniofacial growth cannot be excluded in this adolescent patient.

From a clinical perspective, this approach may be particularly relevant in patients presenting with single‐tooth loss associated with severe combined hard‐ and soft‐tissue deficiency, especially in younger individuals where implant placement is contraindicated or associated with increased risk due to ongoing craniofacial growth. It may also be considered in post‐traumatic defects and in cases with insufficient alveolar bone volume for conventional implant therapy.

Conversely, limitations of the approach include the requirement for a suitable donor tooth and adequate tuberosity bone volume, as well as the inherent technique sensitivity of the procedure. In the absence of these conditions, or in cases with compromised vascularity, alternative treatment strategies may be preferable. In addition, biologic complications inherent to dentoalveolar transplantation procedures, including root resorption, ankylosis, pulpal complications, or compromised graft integration, should also be considered when selecting cases for this approach.

This report represents a single clinical case and therefore does not allow generalization of outcomes. Favorable patient‐related factors, including young age, thick gingival phenotype, good oral hygiene, and ongoing dentoalveolar adaptation, may also have contributed to the observed healing and long‐term tissue stability independently of the surgical technique itself. Quantitative assessment of soft‐tissue parameters and esthetic indices was not systematically performed. The inherent limitations of case reports, including potential selection bias and absence of comparative analysis, should be acknowledged.

Future research should focus on prospective clinical studies with standardized outcome measures and long‐term follow‐up to better define the indications, limitations, and clinical performance of the B2S approach. Comparative investigations with conventional autotransplantation and implant‐based rehabilitation may further clarify its role within contemporary treatment strategies.

## 4. Conclusion

Reconstruction of complex defects in the esthetic zone requires treatment strategies that address both hard‐ and soft‐tissue deficiencies while preserving long‐term functional and esthetic stability. The present case demonstrates that transplantation of a combined tooth–bone–soft‐tissue complex within B2S concept may enable simultaneous restoration of the dentoalveolar unit, particularly in clinical situations where conventional implant therapy may be contraindicated or associated with increased risk.

This biologically driven approach has the potential to reduce the need for staged augmentation procedures while supporting favorable functional and esthetic integration. Nevertheless, given the limited clinical evidence currently available and the absence of standardized quantitative outcome assessment, further prospective studies with larger cohorts and longer follow‐up are required to better define indications, long‐term stability, and comparative effectiveness relative to established reconstructive protocols.

## Funding

No funding was received for this manuscript.

## Conflicts of Interest

The authors declare no conflicts of interest.

## Data Availability

The data that support the findings of this study are available from the corresponding author upon reasonable request.
